# Interplay between the (Poly)phenol Metabolome, Gut Microbiome, and Cardiovascular Health in Women: A Cross-Sectional Study from the TwinsUK Cohort

**DOI:** 10.3390/nu15081900

**Published:** 2023-04-14

**Authors:** Yong Li, Yifan Xu, Caroline Le Roy, Jiaying Hu, Claire J. Steves, Jordana T. Bell, Tim D. Spector, Rachel Gibson, Cristina Menni, Ana Rodriguez-Mateos

**Affiliations:** 1 Department of Nutritional Sciences, School of Life Course and Population Sciences, Faculty of Life Sciences and Medicine, King’s College London, London WC2R 2LS, UK; 2 Department of Twin Research and Genetic Epidemiology, School of Life Course and Population Sciences, Faculty of Life Sciences and Medicine, King’s College London, London WC2R 2LS, UK

**Keywords:** urinary metabolites, gut microbiome alpha diversity, genus, cardiovascular risk score

## Abstract

Background: Dietary (poly)phenol consumption is inversely associated with cardiovascular disease (CVD) risk in epidemiological studies, but little is known about the role of the gut microbiome in this relationship. Methods: In 200 healthy females, aged 62.0 ± 10.0 years, from the TwinsUK cohort, 114 individual (poly)phenol metabolites were measured from spot urine using ultra-high-performance liquid chromatography–mass spectrometry. The associations between metabolites, the gut microbiome (alpha diversity and genera), and cardiovascular scores were investigated using linear mixed models adjusting age, BMI, fibre, energy intake, family relatedness, and multiple testing (FDR < 0.1). Results: Significant associations were found between phenolic acid metabolites, CVD risk, and the gut microbiome. A total of 35 phenolic acid metabolites were associated with the Firmicutes phylum, while 5 metabolites were associated with alpha diversity (FDR-adjusted *p* < 0.05). Negative associations were observed between the atherosclerotic CVD (ASCVD) risk score and five phenolic acid metabolites, two tyrosol metabolites, and daidzein with stdBeta (95% (CI)) ranging from −0.05 (−0.09, −0.01) for 3-(2,4-dihydroxyphenyl)propanoic acid to −0.04 (−0.08, −0.003) for 2-hydroxycinnamic acid (FDR-adjusted *p* < 0.1). The genus 5-7N15 in the Bacteroidetes phylum was positively associated with the same metabolites, including 3-(3,5-dihydroxyphenyl)propanoic acid, 3-(2,4-dihydroxyphenyl)propanoic acid, 3-(3,4-dihydroxyphenyl)propanoic acid), 3-hydroxyphenylethanol-4-sulfate, and 4-hydroxyphenylethanol-3-sulfate)(stdBeta (95% CI): 0.23 (0.09, 0.36) to 0.28 (0.15, 0.42), FDR-adjusted *p* < 0.05), and negatively associated with the ASCVD score (stdBeta (95% CI): −0.05 (−0.09, −0.01), FDR-adjusted *p* = 0.02). Mediation analysis showed that genus 5-7N15 mediated 23.8% of the total effect of 3-(3,4-dihydroxyphenyl)propanoic acid on the ASCVD score. Conclusions: Coffee, tea, red wine, and several vegetables and fruits, especially berries, are the most abundant food sources of phenolic acids that have the strongest associations with CVD risk. We found that the gut microbiome, particularly the genus 5-7N15, partially mediates the negative association between urinary (poly)phenols and cardiovascular risk, supporting a key role of the gut microbiome in the health benefits of dietary (poly)phenols.

## 1. Introduction

(Poly)phenols are a large and broad group of plant secondary metabolites widely abundant in our diet [1]. Based on the number of carbon rings and other structural characteristics, (poly)phenols can be classified into several categories, including flavonoids, phenolic acids, stilbenes, lignans, ellagitannins, and other (poly)phenols. They exist broadly in the plant kingdom and are present in almost all plant-based foods and beverages, especially tea, coffee, red wine, fruits, and vegetables [2]. Growing evidence suggests that (poly)phenols play a role in reducing the risk of multiple chronic diseases such as cardiovascular disease (CVD) and type 2 diabetes mellitus (T2DM) [3].

Although the mechanisms of action of (poly)phenols in the cardiovascular system are not fully understood, (poly)phenols have been shown to modulate the nitric oxide (NO) pathway to maintain homeostasis in the vascular system [4]. In addition, the inhibition of platelet aggregation, reduction of inflammation, limitation of LDL-C (low-density lipoproteins) oxidation, and improvement of the lipid profile might also contribute to the positive effects of (poly)phenols on cardiometabolic health. Observational and intervention studies have found evidence that (poly)phenols are associated with a decrease in CVD mortality [5], an increase in flow-mediated dilation (FMD), and a reduction in blood pressure (BP) [6].

The absorption, distribution, metabolism, and excretion (ADME) of (poly)phenols have been extensively studied during the last decade [7]. The majority of (poly)phenols are not absorbed in the small intestine but travel to the colon, where they are metabolized by the gut microbiota into simpler and easily absorbed low molecular weight phenolic compounds [7,8]. The effects of (poly)phenol exposure vary among individuals, which can be partially explained by the fact that their absorption partly relies on the metabolism by the gut microbiota [9]. In this bi-directional relationship between (poly)phenols and gut microbiota, the (poly)phenol-induced effects on human health may also be mediated by the antibacterial properties of (poly)phenols [10,11]. (Poly)phenols have been shown to promote the abundance of good bacteria, a prebiotic effect, and can inhibit the growth of certain bacteria, thus affecting the overall composition of the gut microbiota [8].

Research has shown that the high concentration of (poly)phenols inhibits detrimental species growth, for instance, *Enteropathogens Staphylococcus aureus* and *Salmonella typhimurium* have been reported to have a high sensitivity to fruit-derived (poly)phenols [12]. Interventional studies have shown that the consumption of (poly)phenol-rich foods modulates the composition of an individual’s beneficial bacterial community, such as increasing the abundance of *Bifidobacterium*, *Lactobacillus*, *Bacteroides*, and *Anaerostipes* with the consumption of red wine [13,14], berries [15,16,17], or cocoa [18]. Cohort studies have also found an association between gut microbial alpha diversity and red wine consumption, with 20% of the reverse association between body mass index (BMI) and red wine consumption mediated by the effect of red wine consumption on gut microbiota alpha diversity [19]. The composition and diversity of the gut microbiota are strongly correlated with host health and metabolic homeostasis [20]. The beneficial effect of (poly)phenols on host health, therefore, might be triggered by the bi-directional relationship with gut microbiota, including the (poly)phenol metabolising capacity of gut microbes and the impact of (poly)phenols on gut microbiota composition and diversity.

Here we assess whether gut microbial diversity and abundance partially mediates the effect of habitual consumption of dietary (poly)phenols, measured by a validated UHPLC-MS method in urine samples and food frequency questionnaires (FFQs) in 100 female pairs of twins from the TwinsUK cohort.

## 2. Methods

### 2.1. Study Population

This cross-sectional study includes 200 healthy female twins aged 62.0 ± 10.0 (monozygotic twin pairs (MZ) = 45, dizygotic twin pairs (DZ) = 55) from the TwinsUK cohort. This is a national research of voluntary female twins without specific screening criteria to investigate the genetics and heritability of diseases with higher prevalence rate in women [21]. Participants completed spot urine and faecal sample collection, cardiovascular measurement, and FFQ data collection. Informed written consent was provided by all twins. The ethical approval for this study was given by the NHS Research Ethics Committee at the Department of Twin Research and Genetic Epidemiology, King’s College London (the Healthy Ageing Twin Study (H.A.T.S) 07/H0802/84) and the NRES Committee London-Westminster (Flora Twin Study reference 12/LO/0227).

### 2.2. Analysis of (Poly)phenol Metabolites in Urine Using UHPLC-MS

The 200 spot urine samples were processed and analysed following a validated method using micro-elution solid phase extraction coupled with ultra-high-performance liquid chromatography–triple quadrupole mass spectrometry (UHPLC-Q-q-Q MS) and 114 (poly)phenol metabolites were identified and quantified by authentic standards [21]. Briefly, the urine samples were diluted with HPLC water Sigma-Aldrich, Steinheim, Germany) to reach 5-fold dilution before they were acidified with 4% phosphoric acid acidified (85% HPLC grade, Yorlab, Fluka, York, UK) in the equivalent volume. Acidified samples (600 µL) were loaded onto the Oasis 96-well reversed-phase HLB µ-SPE plate (Waters, Eschborn, Germany) and washed with HPLC water and 0.2% acetic acid (200 µL each) (glacial HPLC grade, Thermo Fisher Scientific, Loughborough, UK)) before being eluted with 90 µL of methanol (HPLC grade, Sigma-Aldrich, Steinheim, Germany) containing 0.1% formic acid and 10 nM ammonium formate (HPLC grade, Sigma-Aldrich, Steinheim, Germany). A SHIMADZU 8060 UHPLC-Q-q-Q MS was used to analyse the urine samples for (poly)phenol metabolites (Shimadzu, Kyoto, Japan). An aliquot of 5 µL of sample was injected through a 2.1 × 50 mm, 1.8 µm Raptor Biphenyl Column paired with a 5 × 2.1 mm, 2.7 m guard column (Restek, Bellefonte, PA, USA). With the mobile phases made up of water (phase A) and acetonitrile (phase B), both of which were acidified with 0.1% formic acid, the reverse-phase chromatography was carried out at a flow rate of 0.5 mL/min. The detailed parameters of MS and UPLC followed the published method [21]. A pooled urine sample was made and loaded onto two wells of the µ-SPE plate, of which one was fortified with a mix of target analytes. The pooled and fortified pooled samples were applied as quality controls and helped to calculate the recovery rate. The LabSolutions software (SHIMADZU, Kyoto, Japan) was used for raw data analysis and calculation. The urinary creatinine levels were measured by Affinity Biomarker Labs (London, UK) using the Jaffe method and the concentrations of the metabolites (nM) were adjusted by the creatinine levels (mg/L) into mmol/g creatinine.

### 2.3. Dietary (Poly)phenol Intake Assessment via FFQs

Participants completed the validated European Prospective Investigation into Diet and Cancer (EPIC) Norfolk FFQ [22] (available *n* = 198). The online open access Phenol-Explorer database [23], USDA database, and several published papers [24,25,26,27,28,29,30,31,32,33,34,35,36,37,38,39,40,41,42,43,44,45,46] were used to establish a home database in order to estimate the (poly)phenol intake from each food item listed in the FFQ. Data from the normal phase high performance liquid chromatography (HPLC) methods, chromatography and chromatography after hydrolysis, were selected. (Poly)phenol content data of compounds with sugar moiety were transformed into the corresponding amount of aglycones to be summarized with data from other sources. The procyanidin data analysed by normal phase HPLC were applied first, and the data from chromatography were applied when no data from the normal phase HPLC method were available. As for cooked foods, if only the raw data food source was available, the processed yield factor from Phenol-Explorer database multiplied by the unprocessed raw food content was applied to determine the (poly)phenol content of cooked processed foods. If no yield factor was available, a factor of a similar food item or similar processing method of the same item was applied instead. The calculation of each (poly)phenol content (mg/d) was calculated by the food intake (g/d) multiplied by the corresponding (poly)phenol intake from the home database (mg/100 g) and divided by 100. Total and subclasses of (poly)phenols, followed by the classification of Phenol-Explorer, were calculated by summing up all compounds within the group.

### 2.4. Gut Microbiome Analysis

Faecal samples from volunteers were analysed using 16s rRNA sequencing [3]. Faecal samples were transported to Cornell University on dry ice for DNA amplification after being taken to a clinical appointment or posted in sealed ice packs at −80 °C. Illumina MiSeq platform was used for the sequence of amplicons. Operational taxonomic units (OTUs) were obtained from the 16s rRNA gene sequencing as described [47]. Alpha diversities were quantified as observed OTU numbers and Shannon diversity after standardized with mean as 0 and SD as 1.

### 2.5. Measurements of Cardiovascular Risk Scores

The ASCVD risk score was used to estimate the CVD risk of the participants aged 40–79, with the algorithms including sex, ethnicity, age, smoking status, TC, BP, and history of diabetes calculated by the online ASCVD risk estimator (http://tools.acc.org/ASCVD-Risk-Estimator-Plus/ (accessed on 15 February 2023)). The score is categorized into four levels: low risk; low, borderline risk, intermediate risk, and high risk (<5%; 5–7.4%; 7.5–19.9%, and ≥20%) [48]. This score estimates the risk of developing hard ASCVD (coronary heart disease (CHD) death, nonfatal myocardial infarction, fatal or nonfatal stroke) in the following ten years [49].

The HeartScore was also used to estimate the 10-year risk of fatal and nonfatal cardiovascular disease events, with the algorithms including sex, age, SBP, TC, HDL, and smoking status calculated by the online HeartScore estimator (https://www.heartscore.org/en_GB/ (accessed on 15 February 2023)). Before calculation, participants are required to choose from the four European risk regions, which are based on age- and sex-standardized CVD mortality rates, from which the United Kingdom of Great Britain is categorized into the low-risk European region. A risk prediction algorithm known as the Systematic COronary Risk Evaluation (SCORE) model is used in this score. To date, SCORE2 (an updated prediction model) [50] and SCORE2-OP (SCORE2-Older Persons) [51] projects are the latest, updated versions of the SCORE model. SCORE2 and SCORE2-OP aim to estimate 10-year fatal and nonfatal CVD risk in individuals in Europe without previous CVD or diabetes aged 40 to 69 years (SCORE2) and aged over 70 years (SCORE2-OP) [50,51].

### 2.6. Assessment of Covariates

The analysis was adjusted for the following covariates: family relatedness, age, body mass index (BMI kg/m^2^), daily energy (kcal/day), and fibre intake (g/day). Information on family relatedness and age were collected from the self-report lifestyle questionnaire [52]. The weight and height of the participants were measured to calculate BMI (weight in kilogram/height meter^2^) following the harmonized protocols conducted by trained nurses at the clinical visits [52]. Daily energy (kcal/d) and fibre intake (g/d) were derived from the FFQ with the FFQ EPIC and Nutrition Tool for Analysis (FETA) software.

### 2.7. Statistical Analysis

Statistical analysis was implemented using R version 3.6.2 [53]. Data distribution was explored graphically and normalized by log transformation for the required parameters in the statistical analysis. The association between dietary (poly)phenol (measured from FFQs or urine samples), the gut microbiome (alpha diversity and genera), and cardiovascular risk scores were explored using linear mixed models (‘lme4’ package in R) with family relatedness as a random intercept. Analyses were adjusted for (i) age, BMI, energy, and fibre intake for the association between (poly)phenol measured from urine and FFQs (explanatory variables) and cardiovascular risk scores (response variables); (ii) age, BMI, energy, and fibre intake for the association between urinary (poly)phenol (explanatory variables) and gut microbiome (response variables); (iii) age, BMI, and fibre intake for the association between the gut microbiome (explanatory variables) and cardiovascular risk scores (response variables).

**Figure 1:**
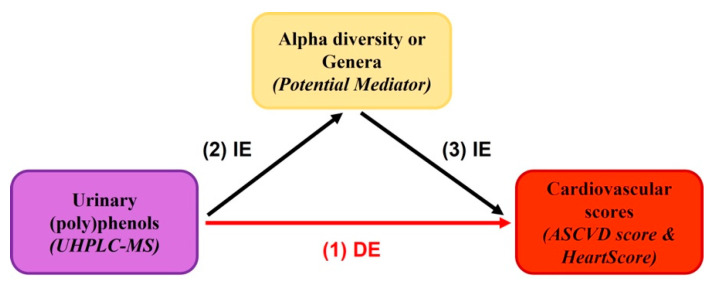
Potential mediation model on the effects of (poly)phenols on cardiovascular risk scores by alpha diversity or genera. (1) Association between circulating (poly)phenol metabolites and cardiovascular scores, adjusted for family relatedness, age, BMI, energy intake, and fibre intake; (2) association between circulating (poly)phenol metabolites and gut microbiome (alpha diversity or genera), adjusted for family relatedness, age, BMI, energy intake, and fibre intake; (3) association between the gut microbiome (alpha diversity or genera) and cardiovascular scores, adjusted for family relatedness, age, BMI, and fibre intake. DE indicates direct effects; IE indicates indirect effects.

Mediation analysis (‘mediation’ package in R) was further assessed to explore the potential mediation effects of the gut microbiome (alpha diversity and genera) on the urinary (poly)phenol metabolites on cardiovascular risk scores. The mediation model quantified both the direct effect of dietary (poly)phenol on cardiovascular scores independent of the gut microbiome (alpha diversity and genera) and the indirect (mediated) effects of (poly)phenol that were mediated by its association with gut microbiome (alpha diversity and genera) (Figure 1). The mediation proportion, calculated by the ratio of indirect-to-total effect, quantifies the significance of the mediator variance and the mediation effect of the microbiota [54]. All analyses were adjusted for multiple testing (Benjamini and Hochberg False Discovery Rate (FDR) < 0.1) [55].

## 3. Results

### 3.1. Population Characteristics

The demographic, alpha diversity, and cardiovascular risk score characteristics of 200 female participants are shown in Table 1. The average age of the participants was 62.0 (SD 10.0) years. Nearly all the subjects were white (99%), with an average BMI of 26.2 kg/m^2^ (SD 4.7). Average fibre and energy intakes were 19.4 g/d (SD 7.0) and 1782.5 kcal/d (SD 545.7), respectively. Alpha diversity was 5.2 (SD 0.7) for Shannon diversity and 336.9 (SD 106.2) for the observed OTU number. On average, their average ASCVD risk score showed an intermediate risk. The contribution of the phyla of this population is described in Supplemental Figure S1. The most abundant phyla included *Proteobacteria* (240 taxa), *Firmicutes* (194 taxa), *Bacteroidetes* (69 taxa), and *Actinobacteria* (66 taxa), which constituted 81.6% of the total gut microbiota.

### 3.2. Urinary (Poly)phenol Metabolites and (Poly)phenol Intake Measured from FFQs

**Figure 2:**
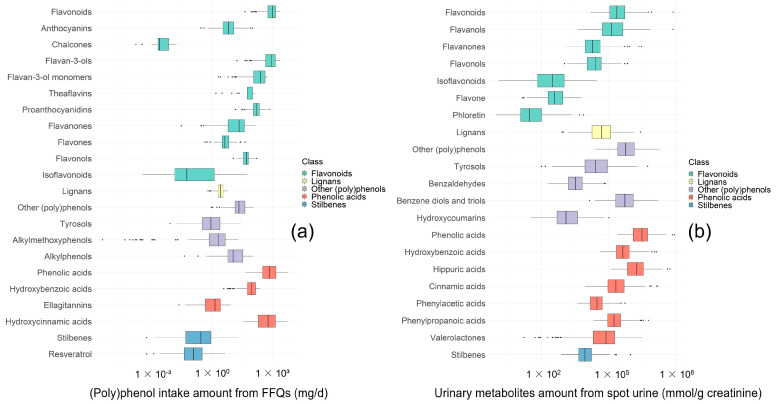
(Poly)phenol intake (mg/d) estimated from FFQs (**a**) and (poly)phenol metabolite levels (mmol/g creatinine) of the spot urine samples (**b**) in this TwinsUK population.

(Poly)phenol intakes estimated from FFQs and urinary (poly)phenol metabolites are presented in Figure 2. The urinary concentration of 114 individual metabolites and the total and subtotal levels of (poly)phenols from different groups are shown in Supplemental Table S1. The average total (poly)phenol metabolite level in the spot urine samples was 7.82 × 10^6^ (SD 1.71 × 10^7^) mmol/g creatinine. The average total (poly)phenol intake measured from FFQs was 2128.9 (SD 961.7) mg/d. Phenolic acids were a major class of (poly)phenols consumed by our population (contributing 49.7% to total (poly)phenol intake measured from FFQs) (Figure 2a), and were the most abundant class of metabolites excreted in urine (Figure 2b), representing 70.6% of the total urinary (poly)phenol metabolites. 

### 3.3. Urinary (Poly)phenol Metabolites and Gut Microbiome Diversity and Composition

The association between urinary (poly)phenol metabolites and alpha diversity is listed in Supplemental Table S2. Alpha diversity, including Shannon diversity and observed OTU numbers, was positively associated with phenolic acids, lignans, and stilbenes. The phenolic acids class associated with five metabolites showed the highest number of associations, followed by four metabolites from lignans, three metabolites from other (poly)phenols, and two metabolites from flavonoids and stilbenes (all FDR-adjusted *p* < 0.05).

The association with the genera is shown in Supplemental Figures S3 and S4. Supplemental Figure S3 exhibits the association between the class and subclass of metabolites and genera. A total of 22 classes and subclasses of metabolites were positively associated with 34 different genera, of which 14 were from the *Firmicutes* phylum. Thus, the associations between genera in *Firmicutes* and each metabolite were further explored and are shown in Supplemental Figure S4. The class of phenolic acids with 42 metabolites associated with *Firmicutes* ranked highest in the number of significant associations, followed by flavonoids with 17 metabolites, other (poly)phenols with 14 metabolites, lignans with seven metabolites, and stilbenes with five metabolites (all FDR-adjusted *p* < 0.05).

### 3.4. Circulating (Poly)phenol Metabolites and Cardiovascular Risk Scores

**Figure 3:**
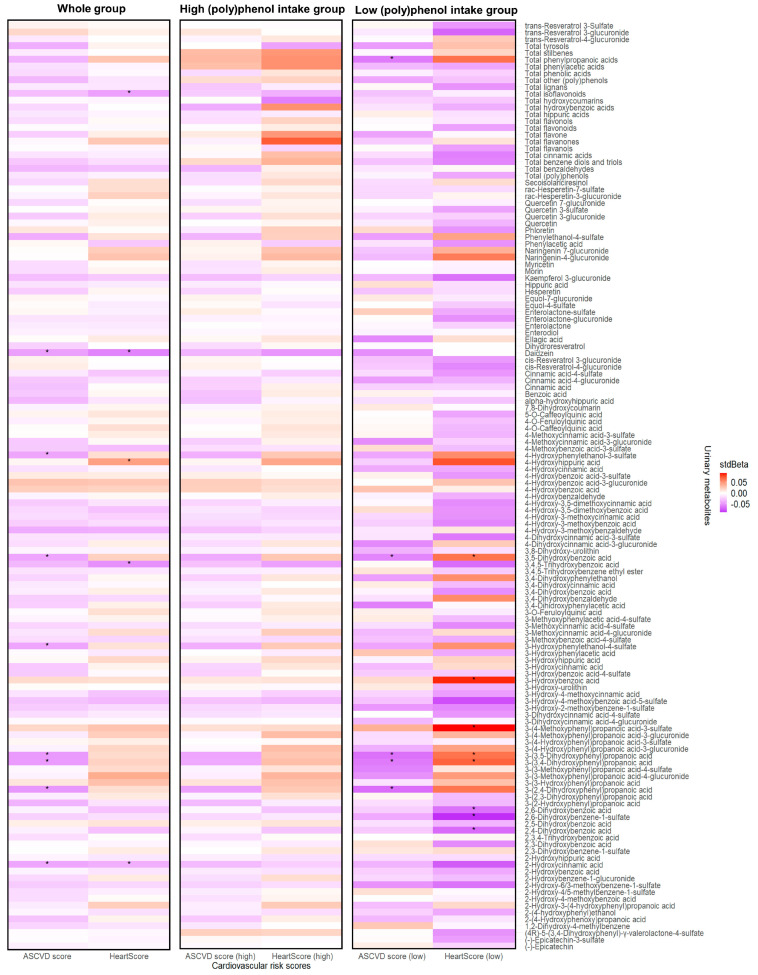
Associations between urinary metabolites and cardiovascular risk scores in the TwinsUK cohort, adjusted for family relatedness, age, BMI, energy intake, and fibre intake for high and low (poly)phenol intake groups. (Poly)phenol intake was adjusted for the whole group. The colour scale indicates the effect (stdBeta) of each metabolite on cardiovascular risk scores. Red and blue illustrate positive and negative effects, respectively, and colour intensity represents the degree of the effects. The asterisks show significance (*: all FDR-adjusted *p* < 0.1). ASCVD, atherosclerotic cardiovascular disease.

The associations between urinary metabolites and cardiovascular risk scores are shown in Figure 3. Negative associations were observed between ASCVD risk score and five urinary phenolic acid metabolites: 3-(3,5-dihydroxyphenyl)propanoic acid, 3-(2,4-dihydroxyphenyl)propanoic acid, 3-(3,4-dihydroxyphenyl)propanoic acid, 3,5-dihydroxybenzoic acid, and 2-hydroxycinnamic acid, 3-hydroxyphenylethanol-4-sulfate, 4-hydroxyphenylethanol-3-sulfate, and daidzein (stdBeta (95% CI): −0.05 (−0.09, −0.01) to −0.04 (−0.08, −0.003), FDR-adjusted *p*-values < 0.1). As for HeartScore, negative associations were found with 3,4,5-trihydroxybenzoic acid, 2-hydroxycinnamic acid, daidzein, and total isoflavonoids (stdBeta (95% CI): −0.06 (−0.09, −0.02) to −0.04 (−0.07, −0.003), FDR-adjusted *p*-values < 0.1). 

Since the average (poly)phenol intake of this population is 2063.20 mg, which is much higher than the previously estimated intake in the UK or other European countries [56,57,58], we divided the population into high and low (poly)phenol intake groups cut at the median estimated (poly)phenol intake (2063.20 mg), with an average total (poly)phenol intake of 1409.63 ± 478.83 mg and 2862.82 ± 753.66 mg, in the low and high intake group, respectively. In the low (poly)phenol intake group, significant associations were shown between ASCVD risk score and five phenolic acid metabolites (stdBeta (95% CI): −0.07 (−0.12, −0.01) to −0.06 (−0.11, −0.001), all FDR-adjusted *p* < 0.1). No associations were observed in the high (poly)phenol intake group. Negative associations were also found in HeartScore and three phenolic acid metabolites (stdBeta (95% CI): −0.09 (−0.16, −0.02) to −0.07 (−0.12, −0.01), all FDR-adjusted *p* < 0.1).

There were no significant associations between (poly)phenol intake measured from FFQs and cardiovascular risk scores (Supplemental Figure S2).

### 3.5. Urinary Metabolites, Gut Microbiome Composition, and Cardiovascular Risk Score

**Figure 4:**
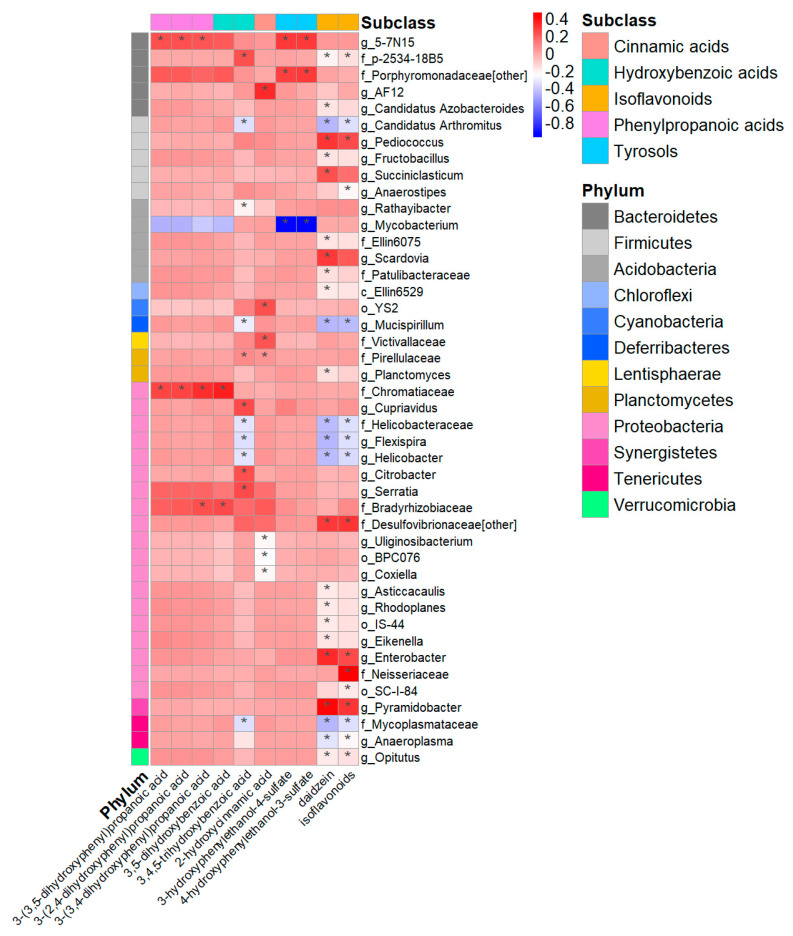
Associations between the significant urinary metabolites in Figure 3 and genera in the TwinsUK cohort, adjusted for family relatedness, age, BMI, energy intake, and fibre intake. The colour scale indicates the effect (stdBeta) of each metabolite on taxa. Red and blue illustrate negative and positive effects, respectively, and colour intensity represents the degree of the effects. This figure only presents the genera with significant associations (*: all FDR-adjusted *p* < 0.05). BMI, body mass index.

The metabolites that were significantly associated with cardiovascular risk scores in the whole group in Figure 3 were selected to test their associations with alpha diversity and genera. No significant associations were found between these metabolites and alpha diversity. The genera with significant associations with urinary (poly)phenols after adjusting for family relatedness, age, BMI, energy, and fibre intake are shown in Figure 4, including 19 genera with positive associations (FDR-adjusted *p* < 0.05). Among the 19 genera, *5-7N15* in the *Bacteroidetes* showed the highest levels and numbers of associations with the metabolites, including all three phenylpropanoic acids (3-(3,5-dihydroxyphenyl)propanoic acid, 3-(2,4-dihydroxyphenyl)propanoic acid, and 3-(3,4-dihydroxyphenyl)propanoic acid), and two tyrosol metabolites (3-hydroxyphenylethanol-4-sulfate and 4-hydroxyphenylethanol-3-sulfate) (stdBeta (95% CI): 0.23 (0.09, 0.36) to 0.28 (0.15, 0.42), FDR-adjusted *p* < 0.05). There were 27 genera negatively associated with the metabolites, and the phylum *Proteobacteria* with 11 genera accounted for the highest among all phyla (all FDR-adjusted *p* < 0.05). 

The genera showing significant associations with urinary (poly)phenol metabolites (in Figure 4) were selected to test the associations with cardiovascular risk scores. The genera with significant negative associations after adjusting for family relatedness, age, BMI, and fibre intake are shown in Table 2, including (i) *5-7N15*, which has the potential ability of the cellulose and hemicellulose [59,60] and (ii) *Scardovia*, one of the seven genera in the *Bifidobacteriaceae* family with high acidogenic and aciduric potential [61]. No significant negative associations were found between the genera in Figure 4 and HeartScore. 

**Figure 5:**
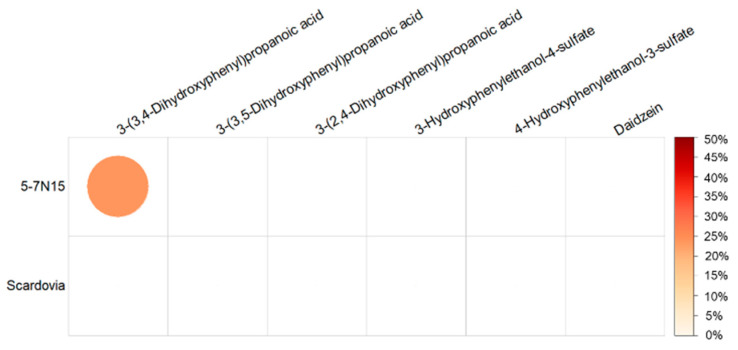
The proportion of the effects of urinary (poly)phenol metabolites on ASCVD risk score mediated via genera. The sequential red colour scale and the circle size indicate the proportion percentage (%) with significance calculated by dividing the indirect effect by the total effect of genera. The red colour intensity represents the degree of the percentage. This figure only presents the genera with significant mediating effect.

A mediation analysis was further conducted to explore the indirect effect of the genera on the relationship between urinary metabolites and ASCVD risk score (Figure 5). The result showed that the genus *5-7N15* in the phylum of *Bacteroidetes* acted as a potential partial mediator in the negative association between ASCVD risk score and the phenolic acid metabolite 3-(3,4-dihydroxyphenyl)propanoic acid (proportion mediated 23.8%, FDR-adjusted *p* < 0.01). 

## 4. Discussion

This work aimed to investigate the associations between circulating (poly)phenol metabolites, the gut microbiome, and cardiovascular disease risk in a subset of women from the TwinsUK cohort. We identified a number of (poly)phenol metabolites that positively correlate with 34 genera, mainly *Firmicutes* and *Bacteroidetes*. Phenolic acid metabolites were the (poly)phenol class that showed the highest levels and numbers of associations with alpha diversity and genera in *Firmicutes*, with five and 42 metabolites associated, respectively. Five phenolic acid metabolites, two tyrosol metabolites, and daidzein showed negative associations with the cardiovascular score. Genus *5-7N15* from the *Bacteroidetes* phylum was positively associated with three phenylpropanoic acid metabolites and two tyrosol metabolites, and negatively associated with the ASCVD score. Genus *5-7N15* showed a potential mediation effect with the (poly)phenol metabolite 3-(3,4-dihydroxyphenyl)propanoic acid on the ASCVD score. Our findings suggest the interaction between (poly)phenols and the gut microbiome might be a potential mechanism on how (poly)phenols can improve cardiovascular health.

Phenolic acids are a major class of dietary (poly)phenols widely abundant in the plant kingdom and our diet [62]. Coffee, tea, red wine, and several vegetables and fruits, especially berries, are the most abundant food sources of phenolic acids [62]. Furthermore, phenolic acids are gut microbial metabolites of nearly all classes of (poly)phenols, for instance, flavonoids [63]. Clearly, phenolic acids are the highest source of (poly)phenols in this study, assessed by FFQs and urine samples. They are hydroxylated derivatives of benzoic, cinnamic, phenylacetic, and phenylpropanoic acids in chemical structure, and evidence suggests they have cardioprotective and anti-atherosclerotic properties [64]. The ASCVD [65] and HeartScore [50,51] risk scores were associated with several subclasses of phenolic acid metabolites, i.e., cinnamic acids (2-hydroxycinnamic acid), benzoic acids (3,5-dihydroxybenzoic acid, 3,4,5-trihydroxybenzoic acid), and three phenylpropanoic acids: (3-(3,5-dihydroxyphenyl)propanoic acid, 3-(2,4-dihydroxyphenyl)propanoic acid, and 3-(3,4-dihydroxyphenyl)propanoic acid). However, these negative associations were only found with the low (poly)phenol intake group, and not in the high intake group, assessed using FFQs. The high intake group had an average total (poly)phenol intake of 2862.82 ± 753.66 mg/d, which is much higher than the estimated intake from the UK or other European countries [56,57,58].

In recent years it has become clear that there is a two-way interaction between (poly)phenols and the gut microbiota. The gut microbiota plays a key role in the biotransformation of (poly)phenols into low-molecular-weight metabolites, while (poly)phenols modulate the gut microbiota by favouring beneficial bacteria growing over pathogenic ones [66]. As only around ten percent of dietary (poly)phenols are absorbed in the small intestine, the microorganisms in the colon transform the remaining (poly)phenols into smaller phenolic acids, including hydroxycinnamic acids, benzoic acids, and phenylpropionic acids [66], which are absorbed and can exert health benefits. In this study, 42 phenolic acid metabolites were associated positively with 32 different genera in the *Firmicutes* phylum and five with two alpha diversity indexes. Benzoic acids in diets may increase intestinal microbiota diversity and promote effective microorganisms, such as *Lactobacillus* and *Bifidobacterium* [67]. Cinnamic acids have shown a microbiota-modulating effect in simulated colonic fermentation and animal models [68]. Here, compared with other classes, phenolic acid metabolites, especially benzoic, cinnamic, and phenylpropanoic acid metabolites, exhibited the closest link with alpha diversity and *Firmicutes*, for instance, *Christensenellaceae* and *Lactobacillaceae*. This is in line with previous research in our lab [17,69]. The favourable effect of phenolic acids on cardiovascular risk scores and the gut microbiome found in this study points to a significant health effect for optimal human health.

Flavonoids, another major class of (poly)phenols [62], also demonstrated beneficial associations with the cardiovascular risk score and the gut microbiome. The chemical structure of daidzein, a phytoestrogen isoflavone, is similar to mammalian estrogens, which enables its protective effect on diseases associated with estrogen control, i.e., cardiovascular disease [70]. In this work, isoflavonoids, particularly daidzein, showed a negative association with the ASCVD score and HeartScore. Interestingly, the main gut microbial metabolites of daidzein, the equol phase II metabolites, were not associated with CVD risk scores. It is well known that there is a high inter-individual variability in equol production and this can affect the health benefits of isoflavone consumption [71]. However, it was not possible to distinguish between equol producers and nonproducers in this study due to its observational nature. We can speculate that other important gut microbial metabolites of daidzein, not investigated in this study, such as ODMA derivatives, may play a role in the association of isoflavones with the CVD risk scores.

Intact circulating flavonoids not metabolized by the gut microbiota may still interact with the gut microbiota and promote gut health by modulating the composition of the gut microbiota, inhibiting pathogens and increasing beneficial genera such as *Bifidobacterium* and *Lactobacillus* [72]. In this research, flavonoids also showed a positive link with nine different genera in *Firmicutes*, for instance, *Lactobacillaceae* and commensal bacteria *Christensenellaceae.* Other potential effects of intact flavonoids include protection of the intestinal barrier function, modulation of the immune system, and modulation of the production of other gut microbial metabolites, such as short-chain fatty acids [73].

Other (poly)phenols were also found with favourable associations, including tyrosol metabolites that were negatively associated with ASCVD risk score, and benzene diols and triols, which were positively associated with Shannon diversity and 19 different genera in *Firmicutes*. The close link between gut microbiota and benzene diols and triols was in line with a previous study in our lab [69]. Moreover, research evidence has shown that hydroxytyrosol, commonly found in olive oil, has anti-inflammatory effects and can improve endothelial function [74]. As for lignans and stilbenes, no links with cardiovascular scores were found. However, the positive association between stilbenes and alpha diversity was in line with a previous gut microbiome study with a subgroup of participants from the same cohort (TwinsUK) [3].

*Firmicutes* and *Bacteroidetes* are the dominant bacterial phyla covering more than 90% of the human gut community [75]. The *Firmicutes* phylum ranked highest in the number of associations with urinary (poly)phenols, with 14 taxa positively associated with the level of 14 different total classes and subclasses of (poly)phenol metabolites. Among the significant taxa associated with urinary (poly)phenol metabolites, *Lachnospiraceae* and *Ruminococcaceae* are abundant families from the *Clostridiales* order that may promote gut health through the production of butyrate, a fermentation product in the colon associated with favourable effects, such as ischemic stroke and metabolic disease [76]. The increase in *Clostridium* and *Christensenellaceae* in the *Clostridiales* order was positively associated with (poly)phenol metabolites, which was in agreement with previous research from our team [17,69]. Evidence has suggested an association between the abundance of *Christensenellaceae* and inflammatory bowel disease [77]. As a key player in human gut health, *Christensenellaceae* has shown significant associations with nearly all (poly)phenol classes, especially phenolic acids. *Bacteroidetes* can break down plant starch and fibre into shorter molecules to provide energy to lean people [78] and contribute to the maintenance of gut health based on their butyrate-producing ability [79]. Research has shown the interplay between a (poly)phenol-rich aronia berry supplement and enriched taxa abundance in the *Bacteroidetes* [17,69]. Similarly, red wine (poly)phenol consumption for a one-month intervention study increased *Bacteroides* abundance [80]. In agreement with the findings above, 16 classes and subclasses of (poly)phenol metabolites were positively associated with eight different genera in the *Bacteroidetes* phylum.

Alterations in the gut microbial community may contribute to cardiovascular disease [81]. Indeed, most established risk factors of CVD, including hypertension, heart failure, and diabetes, are linked with gut dysbiosis [82]. The ability of gut microbes to produce bioactive phenolic metabolites and short-chain fatty acids, including propionate and butyrate, might have a favourable impact on the gut ecosystem, glucose homeostasis, and vascular function [83,84]. Heart failure may also be influenced by the gut microbiota, based on the gut–heart failure hypothesis, suggesting that reduced cardiac output and increased systemic congestion leads to intestinal mucosal ischemia and causes inflammation by elevating bacterial translocation [85]. Other factors such as bowel disease may increase the risk of CVD due to the presence of an abnormal microbial community [82]. In the *Bacteroidetes* phylum, *5-7N15* identified a potential mediation effect on the negative association between 3-(3,4-dihydroxyphenyl)propanoic acid and the ASCVD risk score. *Bacteroidaceae*, the family of *5-7N15*, seems to be involved in nearly all types of human bacterial functional genes in the active gut microbiota. To date, *5-7N15*-related research has been limited to animal models and related to the degradation of cellulose and hemicellulose [59,60], and further analysis of *5-7N15* in the human gut is required. Cellulose, an important part of the wall of plant cells, is a fibre commonly found in plant-based foods, for instance, fruits and vegetables. In this study, although the associations found were adjusted for fibre intake, (poly)phenols represent a group of plant metabolites that are considered a good source of dietary fibre. A (poly)phenol-rich diet might therefore indicate a plant-rich intestine microenvironment for *5-7N15*.

Dietary assessment tools and nutritional biomarkers reflected the (poly)phenol consumption level of the population. FFQ, as a dietary assessment tool, is widely used to quantify dietary (poly)phenol in observational studies [86]. This assessment tool is prone to misreporting bias owing to its self-reported nature, thus limiting its accuracy. A number of (poly)phenol metabolites have been proposed as biomarkers to indicate the consumption of specific (poly)phenols [87] and provide objective information on the exposure levels compared to dietary assessment. Compared with phenolic acids measured from FFQs, which showed no significant associations with cardiovascular scores, phenolic acid metabolites were associated with beneficial effects on cardiovascular scores in the present study. This result is coherent with previous evidence showing that correlations between FFQ and urinary and plasma biomarkers of (poly)phenols were poor [88]. The discrepancies between the two approaches are possibly due to the errors from dietary assessment and individual variability in the absorption and metabolism of ingested (poly)phenols by the digestive system and gut microbiota. Our results suggest that a more objective estimation of (poly)phenol exposure by measuring levels of circulating (poly)phenol metabolites may help to unveil more accurate relationships with health outcomes. However, concerns about biomarkers also exist, such as the reliability of the biomarkers to reflect intake over a long time [87]. A single assessment method would be limited to reflect (poly)phenol consumption levels since each assessment tool has advantages and shortcomings [88]. More accurate and specific tools to reflect habitual (poly)phenol exposure need to be developed.

The novelty of this study lies in the measurement of a wide range of (poly)phenol metabolites from different classes in urine, quantified using authentic standards, which allowed the testing of objective (poly)phenol exposures and associations with gut microbiome and cardiovascular scores. This study is limited by the small sample size, thus restricting the interpretation of the conclusion. It requires further application in a much larger sample to have adequate power. Furthermore, the generalisability of this work is restricted to women and a population of white ethnicity. Further studies are required to investigate the role of sex and different ethnicities in the relationship between (poly)phenol consumption and CVD risk.

## 5. Conclusions

In conclusion, dietary (poly)phenols, in particular phenolic acids, may contribute to lowering CVD risk and improving gut microbiome diversity and composition. Genus *5-7N15* in the *Bacteroidetes* phylum may potentially mediate the inverse association between 3-(3,4-dihydroxyphenyl)propanoic acid and the ASCVD score. Larger cohorts and randomized controlled trials are needed to test whether dietary (poly)phenols can improve cardiovascular health outcomes via the modulation of gut microbiome diversity and composition.

## Figures and Tables

**Table 1:** Demographic Characteristics of the Study Population.

Characteristics	TwinsUK
	n (%)
MZ	45 (45%)
DZ	55 (55%)
White	198 (99%)
	Mean (SD)
Age, yrs	62.0 (10.0)
BMI, kg/m^2^	26.2 (4.7)
Fibre intake, g/d	19.4 (7.0)
Energy intake, kcal	1782.5 (545.7)
Measurements of alpha diversity	
Shannon Diversity	5.2 (0.7)
Observed OTUs number	336.9 (106.2)
Cardiovascular risk scores	
ASCVD risk score	8.1 (9.9)
HeartScore	9.3 (7.5)

MZ, monozygotic; DZ, dizygotic; BMI, body mass index; Observed OTUs number, observed operational taxonomic units number; ASCVD risk score, atherosclerotic cardiovascular disease risk score.

**Table 2:** Cardiovascular score—genera association.

Genera	Cardiovascular Score	stdBeta (95% CI)	FDR-Adjusted *p*-Value
*5-7N15*	ASCVD risk score	−0.05 (−0.09, −0.01)	0.02
*Scardovia*	ASCVD risk score	−0.04 (−0.08, −0.004)	0.02

## Data Availability

The data used in this study are held by the Department of Twin Research at King’s College London. The data can be released to bona fide researchers using our normal procedures overseen by the Wellcome Trust and its guidelines as part of our core funding (https://twinsuk.ac.uk/resources-for-researchers/access-our-data/ (accessed on 10 March 2023)).

## Supplementary Materials

The following supporting information can be downloaded at: https://www.mdpi.com/article/10.3390/nu15081900/s1, Table S1: Concentration of urinary metabolites detected by UHPLC-MS in spot urine sample (mmol/g creatinine). Table S2: Associations between urinary (poly)phenol metabolites and alpha diversity in the TwinsUK cohort, adjusted for family relatedness, age, BMI, energy intake, and fibre intake. Figure S1: The contribution of each phylum. Figure S2: Associations between (poly)phenol intake measured from FFQs and cardiovascular risk scores in the TwinsUK cohort, adjusted for family relatedness, age, BMI, energy intake, and fibre intake. Figure S3: Associations between the class and subclass of metabolites and genera in the TwinsUK cohort, adjusted for family relatedness, age, BMI, energy intake, and fibre intake. Figure S4: Associations between the metabolites and genera in *Firmicutes* in the TwinsUK cohort, adjusted for family relatedness, age, BMI, energy intake, and fibre intake.

## References

[1] Williamson G. (2017). The role of polyphenols in modern nutrition. Nutr. Bull..

[2] Perezjimenez J., Neveu V., Vos F., Scalbert A. (2010). Identification of the 100 richest dietary sources of polyphenols: An application of the phenol-explorer database. Eur. J. Clin. Nutr..

[3] Mompeo O., Spector T.D., Hernandez M.M., Le Roy C., Istas G., Le Sayec M., Mangino M., Jennings A., Rodriguez-Mateos A., Valdes A.M. (2020). Consumption of stilbenes and flavonoids is linked to reduced risk of obesity independently of fiber intake. Nutrients.

[4] Hemler E.C., Hu F.B. (2019). Plant-based diets for cardiovascular disease prevention: All plant foods are not created equal. Curr. Atheroscler. Rep..

[5] Grosso G., Micek A., Godos J., Pajak A., Sciacca S., Galvano F., Giovannucci E.L. (2017). Dietary flavonoid and lignan intake and mortality in prospective cohort studies: Systematic review and dose-response meta-analysis. Am. J. Epidemiol..

[6] Hooper L., Kay C., Abdelhamid A., Kroon P.A., Cohn J.S., Rimm E.B., Cassidy A. (2012). Effects of chocolate, cocoa, and flavan-3-ols on cardiovascular health: A systematic review and meta-analysis of randomized trials. Am. J. Clin. Nutr..

[7] Del Rio D., Rodriguez-Mateos A., Spencer J.P.E., Tognolini M., Borges G., Crozier A. (2013). Dietary (poly)phenolics in human health: Structures, bioavailability, and evidence of protective effects against chronic diseases. Antioxid. Redox Signal..

[8] Rodriguez-Mateos A., Vauzour D., Krueger C.G., Shanmuganayagam D., Reed J., Calani L., Mena P., Del Rio D., Crozier A. (2014). Bioavailability, bioactivity and impact on health of dietary flavonoids and related compounds: An update. Arch. Toxicol..

[9] Rechner A.R., Smith M.A., Kuhnle G., Gibson G.R., Debnam E.S., Srai S.K.S., Moore K.P., Rice-Evans C.A. (2004). Colonic metabolism of dietary polyphenols: Influence of structure on microbial fermentation products. Free Radic. Biol. Med..

[10] Ramírez-Moreno E., Hervert-Hernández D., Sánchez-Mata M., Díez-Marqués C., Goñi I. (2011). Intestinal bioaccessibility of polyphenols and antioxidant capacity of pulp and seeds of cactus pear. Int. J. Food Sci. Nutr..

[11] Fraga C.G., Croft K.D., Kennedy D.O., Tomás-Barberán F.A. (2019). The effects of polyphenols and other bioactives on human health. Food Funct..

[12] Parkar S.G., Stevenson D.E., Skinner M.A. (2008). The potential influence of fruit polyphenols on colonic microflora and human gut health. Int. J. Food Microbiol..

[13] Moreno-Indias I., Sánchez-Alcoholado L., Pérez-Martínez P., Andrés-Lacueva C., Cardona F., Tinahones F.J., Queipo-Ortuño M.I. (2016). Red wine polyphenols modulate fecal microbiota and reduce markers of the metabolic syndrome in obese patients. Food Funct..

[14] Barroso E., Muñoz-González I., Jiménez E., Bartolomé B., Moreno-Arribas M.V., Peláez C., del Carmen Martínez-Cuesta M., Requena T. (2017). Phylogenetic profile of gut microbiota in healthy adults after moderate intake of red wine. Mol. Nutr. Food Res..

[15] Rodriguez-Mateos A., Rendeiro C., Bergillos-Meca T., Tabatabaee S., George T.W., Heiss C., Spencer J.P. (2013). Intake and time dependence of blueberry flavonoid-induced improvements in vascular function: A randomized, controlled, double-blind, crossover intervention study with mechanistic insights into biological activity. Am. J. Clin. Nutr..

[16] Rodriguez-Mateos A., Feliciano R.P., Boeres A., Weber T., dos Santos C.N., Ventura M.R., Heiss C. (2016). Cranberry (poly)phenol metabolites correlate with improvements in vascular function: A double-blind, randomized, controlled, dose-response, crossover study. Mol. Nutr. Food Res..

[17] Istas G., Wood E., Le Sayec M., Rawlings C., Yoon J., Dandavate V., Cera D., Rampelli S., Costabile A., Fromentin E. (2019). Effects of aronia berry (poly)phenols on vascular function and gut microbiota: A double-blind randomized controlled trial in adult men. Am. J. Clin. Nutr..

[18] Tzounis X., Rodriguez-Mateos A., Vulevic J., Gibson G.R., Kwik-Uribe C., Spencer J.P. (2011). Prebiotic evaluation of cocoa-derived flavanols in healthy humans by using a randomized, controlled, double-blind, crossover intervention study. Am. J. Clin. Nutr..

[19] Le Roy C.I., Wells P.M., Si J., Raes J., Bell J., Spector T.D. (2020). Red wine consumption associated with increased gut microbiota α-diversity in 3 independent cohorts. Gastroenterology.

[20] Nicholson J.K., Holmes E., Wilson I.D. (2005). Gut microorganisms, mammalian metabolism and personalized health care. Nat. Rev. Genet..

[21] Domínguez-Fernández M., Xu Y., Yang P.Y.T., Alotaibi W., Gibson R., Hall W.L., Barron L., Ludwig I.A., Cid C., Rodriguez-Mateos A. (2020). Quantitative assessment of dietary (poly)phenol intake: A high-throughput targeted metabolomics method for blood and urine samples. J. Agric. Food Chem..

[22] Bingham S.A., Welch A.A., McTaggart A., Mulligan A.A., Runswick S.A., Luben R., Oakes S., Khaw K.T., Wareham N., Day N.E. (2001). Nutritional methods in the european prospective investigation of cancer in Norfolk. Public Health Nutr..

[23] Phenol-Explorer Database. http://phenol-explorer.eu/.

[24] Bhagwat S., Haytowitz D.B. (2015). USDA Database for the Isoflavone Content of Selected Foods. https://data.nal.usda.gov/dataset/usda-database-isoflavone-content-selected-foods-release-21-november-2015.

[25] Bhagwat S., Haytowitz D.B. (2015). USDA Database for the Proanthocyanidin Content of Selected Foods. https://data.nal.usda.gov/dataset/usda-database-proanthocyanidin-content-selected-foods-release-2-2015.

[26] Bhagwat S., Haytowitz D.B. (2016). USDA Database for the Flavonoid Content of Selected Foods. https://data.nal.usda.gov/dataset/usda-database-flavonoid-content-selected-foods-release-32-november-2015.

[27] Alonso-Esteban J.I., Pinela J., Ćirić A., Calhelha R.C., Soković M., Ferreira I.C., Barros L., Torija-Isasa E., Sánchez-Mata M.D.C. (2021). Chemical composition and biological activities of whole and dehulled hemp (*Cannabis sativa* L.) seeds. Food Chem..

[28] Alvarez-Jubete L., Wijngaard H., Arendt E., Gallagher E. (2010). Polyphenol composition and in vitro antioxidant activity of amaranth, quinoa buckwheat and wheat as affected by sprouting and baking. Food Chem..

[29] Bertin R.L., Gonzaga L.V., Borges G.D.S.C., Azevedo M.S., Maltez H.F., Heller M., Micke G.A., Tavares L.B.B., Fett R. (2014). Nutrient composition and, identification/quantification of major phenolic compounds in *Sarcocornia ambigua* (Amaranthaceae) using HPLC–ESI-MS/MS. Food Res. Int..

[30] Cai H., Zhang Q., Shen L., Luo J., Zhu R., Mao J., Zhao M., Cai C. (2019). Phenolic profile and antioxidant activity of Chinese rice wine fermented with different rice materials and starters. LWT—Food Sci. Technol..

[31] Carvalho A.V., da Silveira T.F.F., Mattietto R.D.A., de Oliveira M.D.S.P., Godoy H.T. (2016). Chemical composition and antioxidant capacity of açaí (*Euterpe oleracea*) genotypes and commercial pulps. J. Sci. Food Agric..

[32] Cicero N., Albergamo A., Salvo A., Bua G.D., Bartolomeo G., Mangano V., Rotondo A., Di Stefano V., Di Bella G., Dugo G. (2018). Chemical characterization of a variety of cold-pressed gourmet oils available on the Brazilian market. Food Res. Int..

[33] Gao Q.-H., Wu C.-S., Yu J.-G., Wang M., Ma Y.-J., Li C.-L. (2012). Textural characteristic, antioxidant activity, sugar, organic acid, and phenolic profiles of 10 promising jujube (*Ziziphus jujuba* Mill.) selections. J. Food Sci..

[34] Gundogdu M. (2013). Determination of antioxidant capacities and biochemical compounds of *Berberis vulgaris* L. fruits. Adv. Environ. Biol..

[35] Hassan M.A., Xu T., Tian Y., Zhong Y., Ali F.A.Z., Yang X., Lu B. (2021). Health benefits and phenolic compounds of *Moringa oleifera* leaves: A comprehensive review. Phytomedicine.

[36] Karunasiri A.N., Gunawardane M., Senanayake C.M., Jayathilaka N., Seneviratne K.N. (2020). Antioxidant and nutritional properties of domestic and commercial coconut milk preparations. Int. J. Food Sci..

[37] Kašpar M., Bajer T., Bajerová P., Česla P. (2022). Comparison of phenolic profile of balsamic vinegars determined using liquid and gas chromatography coupled with mass spectrometry. Molecules.

[38] Kim M.-Y., Seguin P., Ahn J.-K., Kim J.-J., Chun S.-C., Kim E.-H., Seo S.-H., Kang E.-Y., Kim S.-L., Park Y.-J. (2008). Phenolic compound concentration and antioxidant activities of edible and medicinal mushrooms from Korea. J. Agric. Food Chem..

[39] Lv Q., Luo F., Zhao X., Liu Y., Hu G., Sun C., Li X., Chen K. (2015). Identification of proanthocyanidins from litchi (*Litchi chinensis* Sonn.) pulp by LC-ESI-Q-TOF-MS and their antioxidant activity. PLoS ONE.

[40] Miceli N., Trovato A., Marino A., Bellinghieri V., Melchini A., Dugo P., Cacciola F., Donato P., Mondello L., Güvenç A. (2011). Phenolic composition and biological activities of *Juniperus drupacea* Labill. berries from Turkey. Food Chem. Toxicol..

[41] Mocan A., Cairone F., Locatelli M., Cacciagrano F., Carradori S., Vodnar D.C., Crisan G., Simonetti G., Cesa S. (2019). Polyphenols from *Lycium barbarum* (Goji) fruit european cultivars at different maturation steps: Extraction, HPLC-DAD analyses, and biological evaluation. Antioxidants.

[42] Muala W.C.B., Desobgo Z.S.C., Jong N.E. (2021). Optimization of extraction conditions of phenolic compounds from *Cymbopogon citratus* and evaluation of phenolics and aroma profiles of extract. Heliyon.

[43] Prasanthi P.S., Naveena N., Rao M.V., Bhaskarachary K. (2017). Compositional variability of nutrients and phytochemicals in corn after processing. J. Food Sci. Technol..

[44] Rahman J., de Camargo A.C., Shahidi F. (2017). Phenolic and polyphenolic profiles of chia seeds and their in vitro biological activities. J. Funct. Foods.

[45] Rueda A., Samaniego-Sánchez C., Olalla M., Giménez R., Cabrera-Vique C., Seiquer I., Lara L. (2016). Combination of analytical and chemometric methods as a useful tool for the characterization of extra virgin argan oil and other edible virgin oils. Role of polyphenols and tocopherols. J. AOAC Int..

[46] Xu L., Du B., Xu B. (2015). A systematic, comparative study on the beneficial health components and antioxidant activities of commercially fermented soy products marketed in China. Food Chem..

[47] Jackson M.A., Bell J.T., Spector T.D., Steves C.J. (2016). A heritability-based comparison of methods used to cluster 16S rRNA gene sequences into operational taxonomic units. PeerJ.

[48] Mompeo O., Berry S.E., Spector T.D., Menni C., Mangino M., Gibson R. (2020). Differential associations between *a priori* diet quality scores and markers of cardiovascular health in women: Cross-sectional analyses from TwinsUK. Br. J. Nutr..

[49] Lloyd-Jones D.M., Braun L.T., Ndumele C.E., Smith S.C., Sperling L.S., Virani S.S., Blumenthal R.S. (2018). Use of risk assessment tools to guide decision-making in the primary prevention of atherosclerotic cardiovascular disease: A special report from the american heart association and American college of cardiology. J. Am. Coll. Cardiol..

[50] SCORE2 Working Group and ESC Cardiovascular Risk Collaboration (2021). SCORE2 risk prediction algorithms: New models to estimate 10-year risk of cardiovascular disease in Europe. Eur. Heart J..

[51] SCORE2-OP Working Group and ESC Cardiovascular Risk Collaboration (2021). SCORE2-OP risk prediction algorithms: Estimating incident cardiovascular event risk in older persons in four geographical risk regions. Eur. Heart J..

[52] Verdi S., Abbasian G., Bowyer R.C.E., Lachance G., Yarand D., Christofidou P., Mangino M., Menni C., Bell J.T., Falchi M. (2019). TwinsUK: The UK adult twin registry update. Twin Res. Hum. Genet..

[53] R Core Team (2022). R: A Language and Environment for Statistical Computing.

[54] Imai K., Keele L., Tingley D., Yamamoto T., Vinod H.D. (2010). Advances in social science research using R, chap. Causal Mediation Analysis Using R.

[55] Li J., Ji L. (2005). Adjusting multiple testing in multilocus analyses using the eigenvalues of a correlation matrix. Heredity.

[56] Londoño C., Cayssials V., de Villasante I., Crous-Bou M., Scalbert A., Weiderpass E., Agudo A., Tjønneland A., Olsen A., Overvad K. (2021). Polyphenol intake and epithelial ovarian cancer risk in the european prospective investigation into cancer and nutrition (EPIC) study. Antioxidants.

[57] Zamora-Ros R., Knaze V., Rothwell J.A., Hémon B., Moskal A., Overvad K., Tjønneland A., Kyrø C., Fagherazzi G., Boutron-Ruault M.-C. (2015). Dietary polyphenol intake in Europe: The European prospective investigation into cancer and nutrition (EPIC) study. Eur. J. Nutr..

[58] Ziauddeen N., Rosi A., Del Rio D., Amoutzopoulos B., Nicholson S., Page P., Scazzina F., Brighenti F., Ray S., Mena P. (2018). Dietary intake of (poly)phenols in children and adults: Cross-sectional analysis of UK national diet and nutrition survey rolling programme (2008–2014). Eur. J. Nutr..

[59] Huang S., Ji S., Yan H., Hao Y., Zhang J., Wang Y., Cao Z., Li S. (2020). The day-to-day stability of the ruminal and fecal microbiota in lactating dairy cows. MicrobiologyOpen.

[60] Li Y., Li X., Liu Y., Nie C., Chen C., Niu J., Zhang W. (2022). Comparison of bacterial and fungal community structure and potential function analysis of yak feces before and after weaning. Bio. Med. Res. Int..

[61] Kameda M., Abiko Y., Washio J., Tanner A.C.R., Kressirer C.A., Mizoguchi I., Takahashi N. (2020). Sugar metabolism of *Scardovia wiggsiae*, a novel caries-associated bacterium. Front. Microbiol..

[62] Zamora-Ros R., Rothwell J.A., Scalbert A., Knaze V., Romieu I., Slimani N., Fagherazzi G., Perquier F., Touillaud M., Molina-Montes E. (2013). Dietary intakes and food sources of phenolic acids in the European prospective investigation into cancer and nutrition (EPIC) study. Br. J. Nutr..

[63] Lavefve L., Howard L.R., Carbonero F. (2019). Berry polyphenols metabolism and impact on human gut microbiota and health. Food Funct..

[64] Afnan, Saleem A., Akhtar M.F., Sharif A., Akhtar B., Siddique R., Ashraf G.M., Alghamdi B.S., Alharthy S.A. (2022). Anticancer, cardio-protective and anti-inflammatory potential of natural-sources-derived phenolic acids. Molecules.

[65] Alborzi A., Attar A., Sayadi M., Nouri F. (2021). The effects of intensive blood pressure control on cardiovascular outcomes based on 10-year ASCVD risk score: An analysis of a clinical trial. Cardiol. Res. Pract..

[66] Selma M.V., Espiín J.C., Tomás-Barberán F.A. (2009). Interaction between Phenolics and gut microbiota: Role in human health. J. Agric. Food Chem..

[67] Mao X., Yang Q., Chen D., Yu B., He J. (2019). Benzoic acid used as food and feed additives can regulate gut functions. Bio. Med. Res. Int..

[68] Leonard W., Zhang P., Ying D., Fang Z. (2020). Hydroxycinnamic acids on gut microbiota and health. Compr. Rev. Food Sci. Food Saf..

[69] Le Sayec M., Xu Y., Laiola M., Gallego F.A., Katsikioti D., Durbidge C., Kivisild U., Armes S., Lecomte M., Fança-Berthon P. (2022). The effects of *Aronia berry* (poly)phenol supplementation on arterial function and the gut microbiome in middle aged men and women: Results from a randomized controlled trial. Clin. Nutr..

[70] Alshehri M.M., Sharifi-Rad J., Herrera-Bravo J., Jara E.L., Salazar L.A., Kregiel D., Uprety Y., Akram M., Iqbal M., Martorell M. (2021). Therapeutic potential of isoflavones with an emphasis on daidzein. Oxidative Med. Cell Longev..

[71] Lampe J.W. (2009). Is equol the key to the efficacy of soy foods?. Am. J. Clin. Nutr..

[72] Pei R., Liu X., Bolling B. (2020). Flavonoids and gut health. Curr. Opin. Biotechnol..

[73] Xiong H.-H., Lin S.-Y., Chen L.-L., Ouyang K.-H., Wang W.-J. (2023). The interaction between flavonoids and intestinal microbes: A review. Foods.

[74] Borzì A.M., Biondi A., Basile F., Luca S., Vicari E.S.D., Vacante M. (2018). Olive oil effects on colorectal cancer. Nutrients.

[75] Magne F., Gotteland M., Gauthier L., Zazueta A., Pesoa S., Navarrete P., Balamurugan R. (2020). The firmicutes/bacteroidetes ratio: A relevant marker of gut dysbiosis in obese patients?. Nutrients.

[76] Canani R.B., Di Costanzo M., Leone L., Pedata M., Meli R., Calignano A. (2011). Potential beneficial effects of butyrate in intestinal and extraintestinal diseases. World J. Gastroenterol..

[77] Waters J.L., Ley R.E. (2019). The human gut bacteria Christensenellaceae are widespread, heritable, and associated with health. BMC Biol..

[78] Sarojini S. (2018). Diet, Microbiome and Health.

[79] Thomas F., Hehemann J.-H., Rebuffet E., Czjzek M., Michel G. (2011). Environmental and gut bacteroidetes: The food connection. Front. Microbiol..

[80] Queipo-Ortuño M.I., Boto-Ordóñez M., Murri M., Gomez-Zumaquero J.M., Clemente-Postigo M., Estruch R., Cardona Diaz F., Andrés-Lacueva C., Tinahones F.J. (2012). Influence of red wine polyphenols and ethanol on the gut microbiota ecology and biochemical biomarkers. Am. J. Clin. Nutr..

[81] Trøseid M., Andersen G.Ø., Broch K., Hov J.R. (2020). The gut microbiome in coronary artery disease and heart failure: Current knowledge and future directions. EBioMedicine.

[82] Astudillo A.A., Mayrovitz H.N. (2021). The gut microbiome and cardiovascular disease. Cureus.

[83] Tomás-Barberán F.A., Selma M.V., Espín J.C. (2016). Interactions of gut microbiota with dietary polyphenols and consequences to human health. Curr. Opin. Clin. Nutr. Metab. Care.

[84] Chambers E.S., Preston T., Frost G., Morrison D.J. (2018). Role of gut microbiota-generated short-chain fatty acids in metabolic and cardiovascular health. Curr. Nutr. Rep..

[85] Sandek A., Bauditz J., Swidsinski A., Buhner S., Weber-Eibel J., von Haehling S., Schroedl W., Karhausen T., Doehner W., Rauchhaus M. (2007). Altered intestinal function in patients with chronic heart failure. J. Am. Coll. Cardiol..

[86] Xu Y., Le Sayec M., Roberts C., Hein S., Rodriguez-Mateos A., Gibson R. (2021). Dietary assessment methods to estimate (poly)phenol intake in epidemiological studies: A systematic review. Adv. Nutr. Int. Rev. J..

[87] Spencer J.P.E., El Mohsen M.M.A., Minihane A.-M., Mathers J.C. (2007). Biomarkers of the intake of dietary polyphenols: Strengths, limitations and application in nutrition research. Br. J. Nutr..

[88] Xu Y., Li Y., Ma X., Alotaibi W., Le Sayec M., Cheok A., Wood E., Hein S., Yang P.Y.T., Hall W.L. (2023). Comparison between dietary assessment methods and biomarkers in estimating dietary (poly)phenol intake. Food Funct..

