# Supplementary material for: Interplay between the (Poly)phenol Metabolome, Gut Microbiome, and Cardiovascular Health in Women: A Cross-Sectional Study from the TwinsUK Cohort
Source: Nutrients. 2023 Apr 14;15(8):1900. doi: 10.3390/nu15081900 (PMC10141398; doi:10.3390/nu15081900)
Supplement: Supplementary file 1 [file nutrients-15-01900-s001.zip › suppplemental figures.pdf]

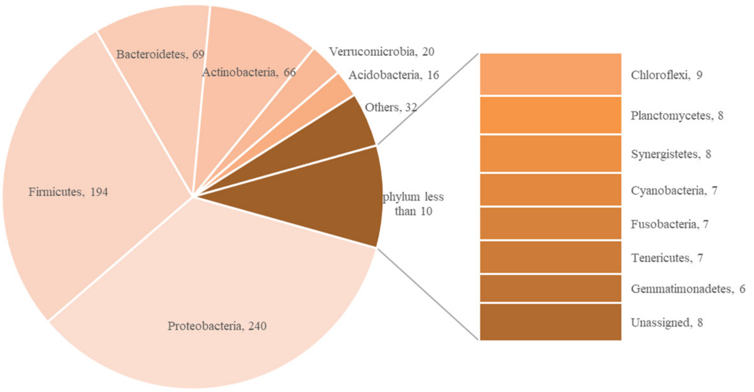

Supplement Figure S1. The contribution of each phylum. The category of *Others* represents the combination of phylum less than 3, including one *FBF, GN02, OD1, SR1, TM6, TM7, WPS-2, Caldithrix, Thermi, Deferribacteres, Fibrobacteres, Crenarchaeota, Euryarchaeota, Armatimonadetes*; two WS3, *Chlamydiae, Chlorobi, Elusimicrobia, Nitrospirae*; and three *Fibrobacteres, Lentisphaerae, Spirochaetes*.

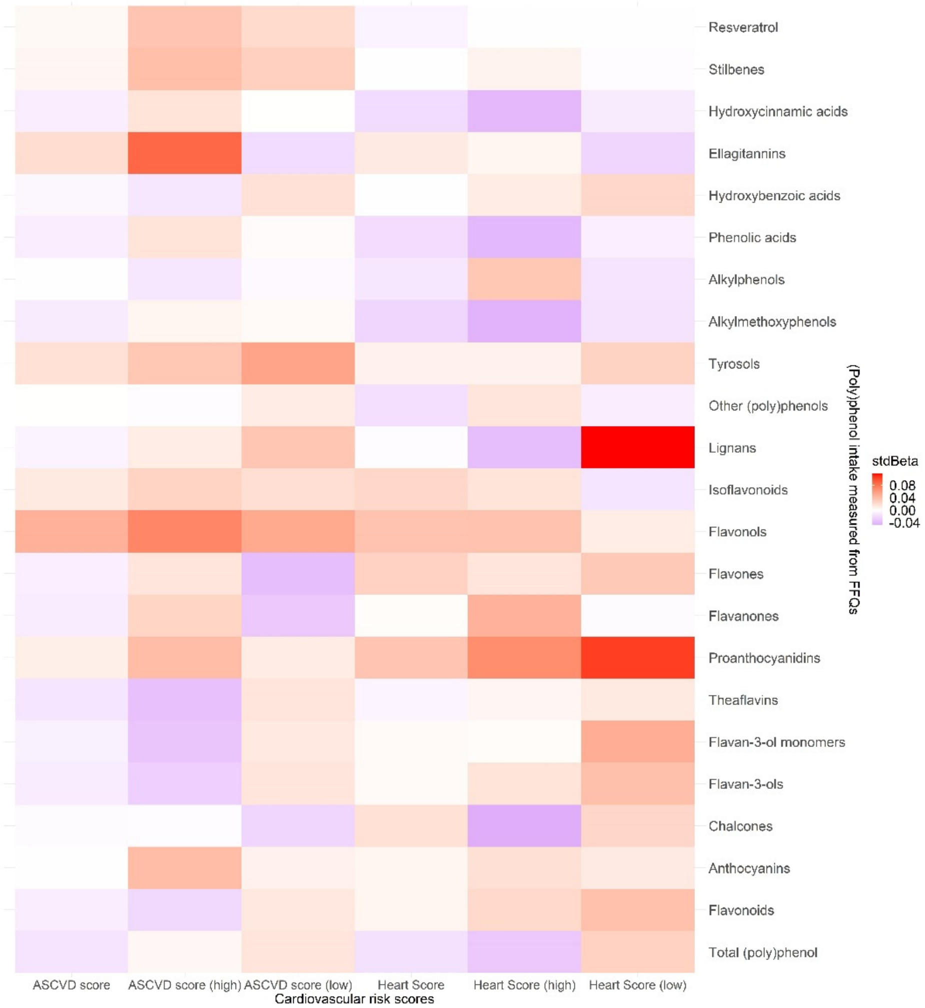

Supplement Figure S2. Associations between (poly)phenol intake measured from FFQs and cardiovascular risk scores in the TwinsUK cohort, adjusted for family relatedness, age, BMI, energy intake, and fibre intake. The colour scale indicates the effect (stdBeta) of each (poly)phenol intake on cardiovascular risk scores. Red and blue illustrate positive, and negative effects and colour intensity represents the degree of the effects. The asterisks showed significance (*: all FDR adjusted $p < 0.05$). ASCVD risk score, atherosclerotic cardiovascular disease risk score; BMI, body mass index; FFQs, food frequency questionnaires.

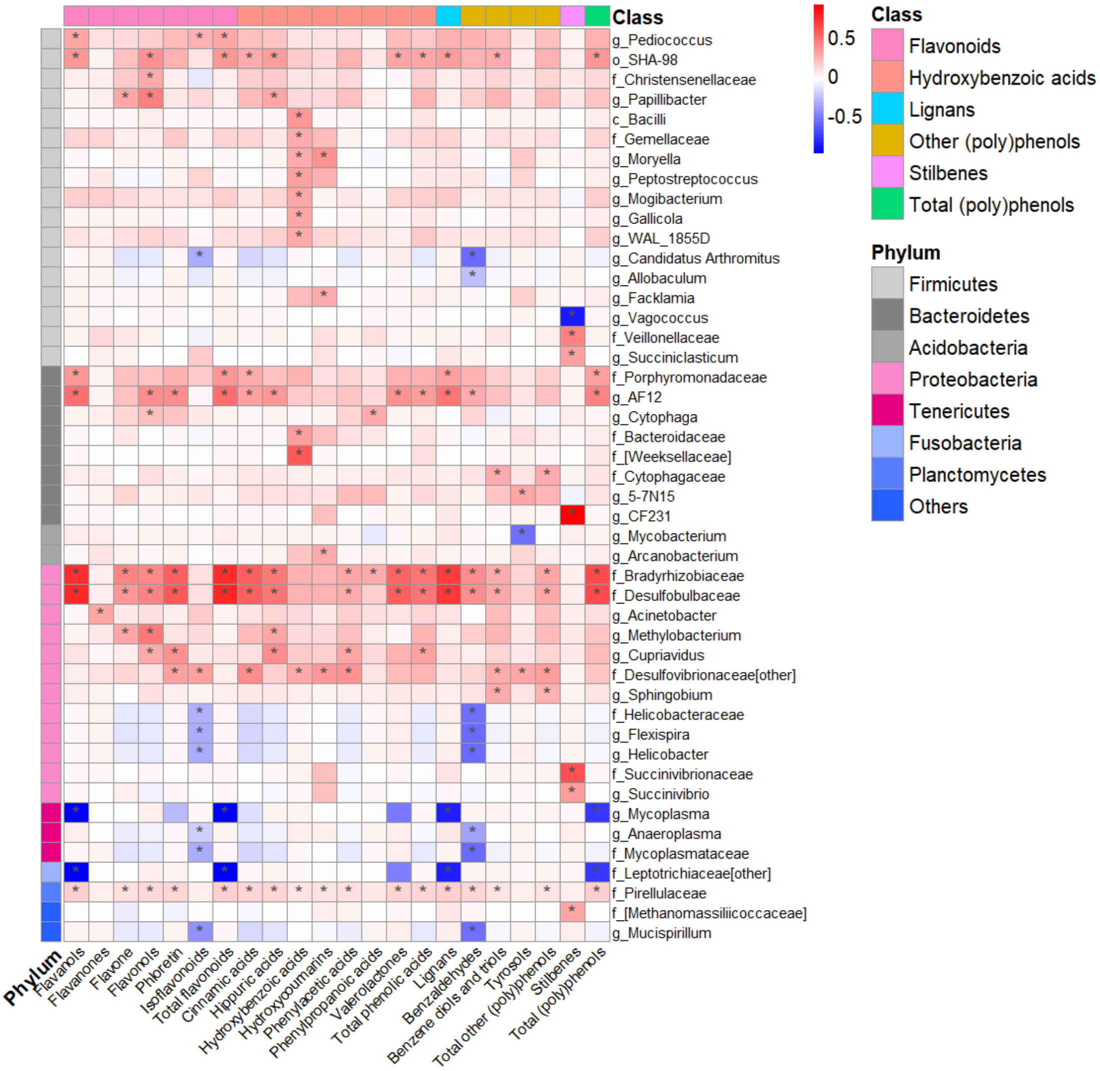

Supplement Figure S3. Associations between the class and subclass of metabolites and genera in the TwinsUK cohort, adjusted for family relatedness, age, BMI, energy intake, and fibre intake. The colour scale indicates the effect (stdBeta) of each subclass of metabolite on the genera. Red and blue illustrate negative and positive effects, and colour intensity represents the degree of the effects. This figure only presents the genera with significant associations (*: all FDR adjusted $p < 0.05$). BMI, body mass index.

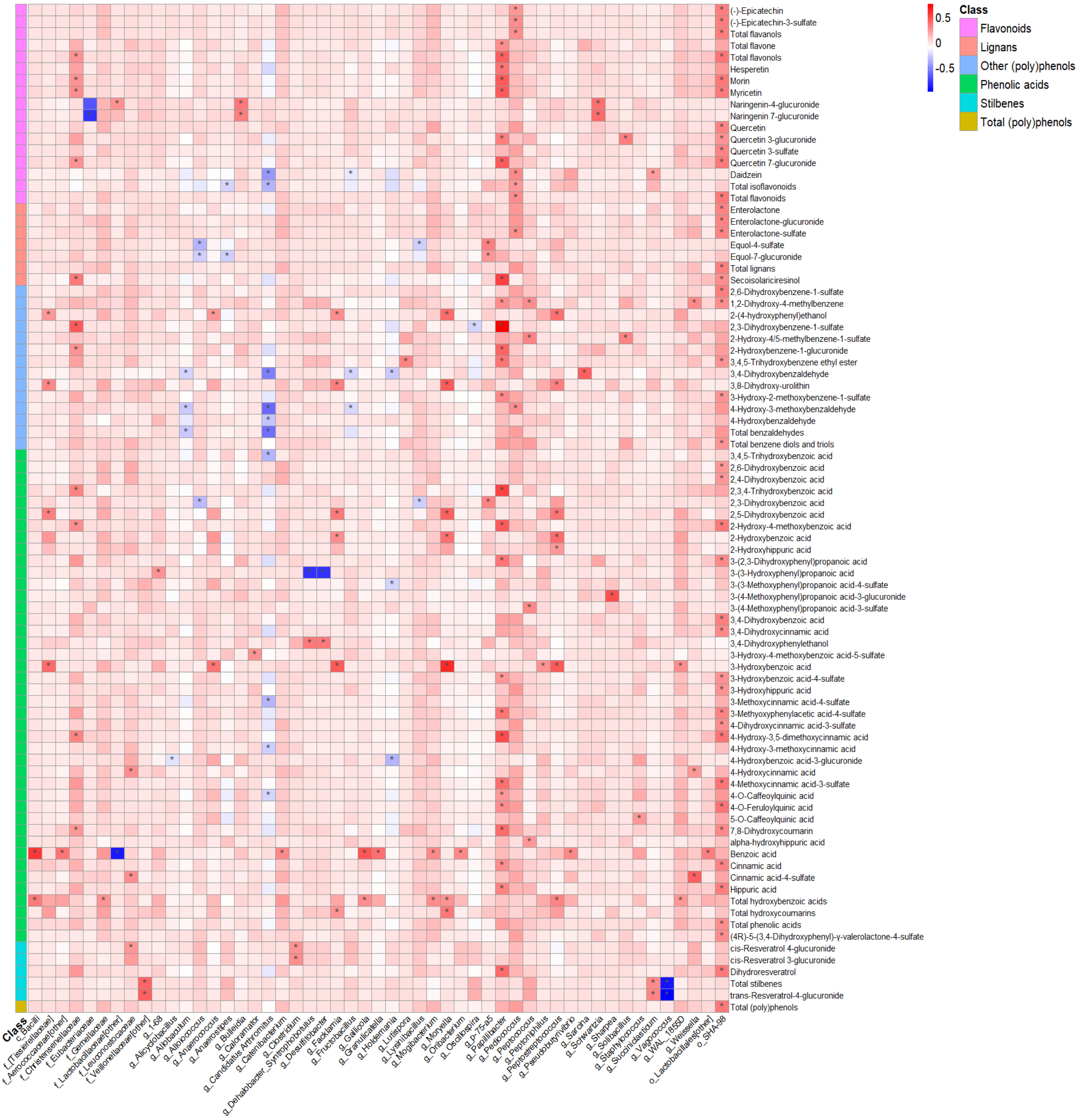

Supplement Figure S4. Associations between the metabolites and genera in *Firmicutes* in the TwinsUK cohort, adjusted for family relatedness, age, BMI, energy intake, and fibre intake. The colour scale indicates the effect (stdBeta) of each subclass of metabolite on the genera. Red and blue illustrate negative and positive effects, and colour intensity represents the degree of the effects. This figure only presents the genera with significant associations (*: all FDR adjusted $p < 0.05$). BMI, body mass index.
